# Conduction-band effective mass and bandgap of ZnSnN_2_ earth-abundant solar absorber

**DOI:** 10.1038/s41598-017-14850-7

**Published:** 2017-11-08

**Authors:** Xiang Cao, Fumio Kawamura, Yoshihiko Ninomiya, Takashi Taniguchi, Naoomi Yamada

**Affiliations:** 1 Department of Applied Chemistry, Chubu University, Kasugai, 487-8501 Japan; 2 National Institute for Materials Science (NIMS), Tsukuba, 305-0044 Japan

## Abstract

Pseudo III-V nitride ZnSnN_2_ is an earth-abundant semiconductor with a high optical absorption coefficient in the solar spectrum. Its bandgap can be tuned by controlling the cation sublattice disorder. Thus, it is a potential candidate for photovoltaic absorber materials. However, its important basic properties such as the intrinsic bandgap and effective mass have not yet been quantitatively determined. This paper presents a detailed optical absorption analysis of disordered ZnSnN_2_ degenerately doped with oxygen (ZnSnN_2−*x*_O_*x*_) in the ultraviolet to infrared region to determine the conduction-band effective mass (*m*_c_^*^) and intrinsic bandgap (*E*_g_). ZnSnN_2−*x*_O_*x*_ epilayers are *n*-type degenerate semiconductors, which exhibit clear free-electron absorption in the infrared region. By analysing the free-electron absorption using the Drude model, *m*_c_^*^ was determined to be (0.37 ± 0.05)*m*_0_ (*m*_0_ denotes the free electron mass). The fundamental absorption edge in the visible to ultraviolet region shows a blue shift with increasing electron density. The analysis of the blue shift in the framework of the Burstein-Moss effect gives the *E*_g_ value of 0.94 ± 0.02 eV. We believe that the findings of this study will provide important information to establish this material as a photovoltaic absorber.

## Introduction

Photovoltaics represent a promising approach to achieving truly sustainable energy, and considerable efforts have been directed toward the development of high-efficiency photovoltaic cells to meet the increasing global energy demand. In photovoltaic cells based on inorganic semiconductors, the conversion efficiency strongly depends on its bandgap. The theoretical limit of efficiency for a single-junction solar cell reaches a maximum value of ~34%, when the bandgap (*E*_g_) of the semiconductor is ~1.4 eV^1^.

Binary semiconductors with an *E*_g_ of approximately 1.4 eV are limited to a few compounds such as GaAs, InP, and CdTe^2^. As for semiconductor alloys, In_*x*_Ga_1−*x*_N is a candidate material for a photovoltaic absorber because the *E*_g_ can be tuned to ~1.4 eV by adjusting the indium content (*x*) to ~0.65^3^. However, these compounds are composed of rare or toxic elements, and it is difficult to produce cost-effective photovoltaic cells based on these semiconductors on large-area substrates. This situation has motivated researchers to seek earth-abundant multicomponent semiconductors with an ideal *E*_g_ value of 1.4 eV.

In 2013, the first-principles study by Lahourcade *et al*. showed that ternary ZnSnN_2_ is an earth-abundant semiconductor with an excellent *E*_g_ of 1.42 eV^4^. Since then, investigation of ZnSnN_2_ as a photovoltaic absorber material has been initiated in recent years. However, ZnSnN_2_ is still not well understood: the synthesis of ZnSnN_2_ thin film was first reported in 2012^5,6^, and the synthesis of ZnSnN_2_ powders was first reported in 2016^7^. Even the lattice constants have been determined only recently^7,8^. Although extensive study on this material has begun, the basic electronic and optical properties have not been fully elucidated yet.

**Figure 1:**
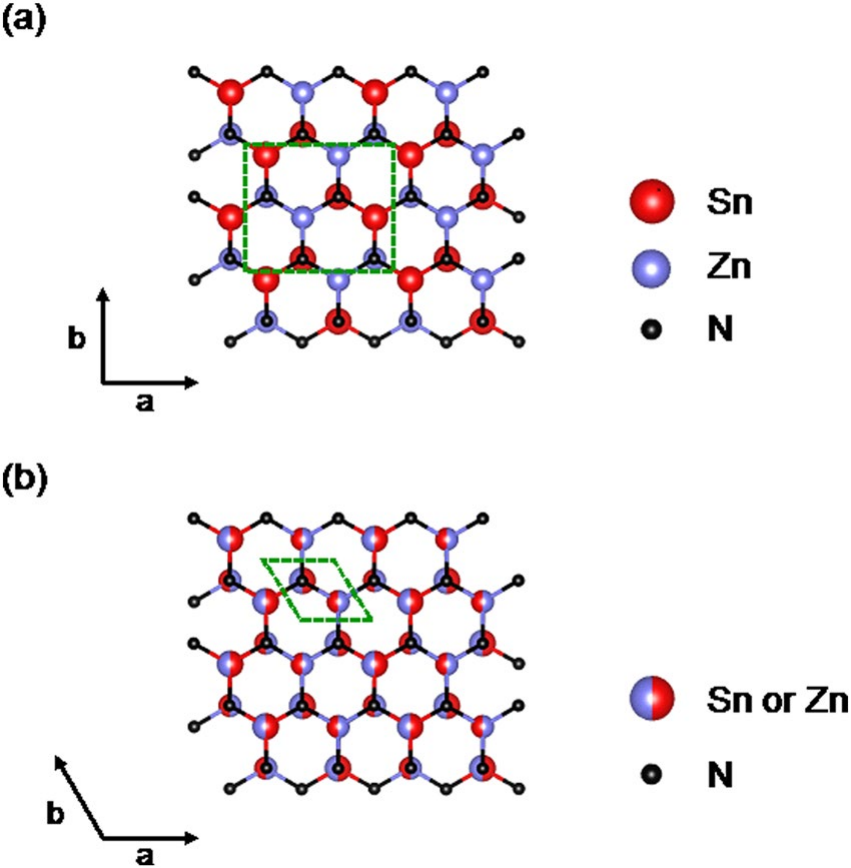
Crystal structures of (**a**) ordered- and (**b**) disordered-phase of ZnSnN_2_ (from [001] direction). The dotted square in (**a**) and rhombus in (**b**) represent the unit cells. The crystal structure was visualized using VESTA software^51^.

ZnSnN_2_ has two phases: ordered-phase and disordered-phase, depending on the cation-sublattice ordering, similar to ZnGeN_2_. The ordered-phase is derived by alternately replacing the cation-sublattice in the wurtzite structure with Zn and Sn (Fig. 1a). This structure is referred to as *β*-NaFeO_2_ structure with orthorhombic symmetry. In the disordered-phase, Zn and Sn randomly occupied the cation-sublattice in the wurtzite-like structure (Fig. 1b). Veal *et al*. conducted the first principle calculations and indicated that the ordered- and disordered-phases have *E*_g_ values of 2.3 and 0.98 eV, respectively^9^. Based on these values, we thought that the disordered ZnSnN_2_ is more ideal for a photovoltaic absorber.

The basic properties of the newly-discovered disordered ZnSnN_2_ have not yet been thoroughly investigated. For instance, effective mass and the intrinsic *E*_g_ of ZnSnN_2_ remain undetermined. These properties are very important for establishing ZnSnN_2_ as a photovoltaic absorber. Insufficient knowledge of the *m*_c_^*^ and *E*_g_ values make it difficult to use this material for photovoltaic absorbers. Although optically determined *E*_g_ values have been reported by some research groups, the values are scattered in the range of 1.0–2.0 eV (Table 1)^9–12^. We hypothesize that the reported values are not incorrect and their scattering originates from the blue shift of *E*_g_ due to the conduction-band filling effect (the so-called Burstein-Moss shift), because the conduction-electron densities were largely different in each study. As will be described later, this hypothesis was justified, and thus the intrinsic *E*_g_ value was successfully determined from the analysis in the framework of the Burstein-Moss effect. As for the reported *m*_c_^*^ values, a large discrepancy between the theoretical and experimental values exists, which are summarized in Table 1. The theoretical study predicted small *m*_c_^*^ values of 0.12*m*_0_ (*m*_0_ denotes the free-electron mass)^9^, whereas the experimental study yielded a value four times larger at 0.5*m*_0_. This large discrepancy motivated us to discover which value is correct. The experimental *m*_c_^*^ value was derived from the analysis of the Burstein-Moss shift^11^. Strictly speaking, the effective mass determined by this method is not *m*_c_^*^ but the ‘reduced’ effective mass (*m*_vc_^*^), as the *m*_vc_^*^ is a function of *m*_c_^*^ and the valence-band effective mass (*m*_v_^*^), as discussed later. The *m*_vc_^*^ value becomes equal to *m*_c_^*^ value only when the *m*_v_^*^ value is much higher than that of *m*_c_^*^ (*m*_c_^*^/*m*_v_^*^ ≈ 0). Thus, it is likely that the reported experimental value deviates somewhat from the true value. To obtain the correct *m*_c_^*^ value, we performed an analysis of the free-electron absorption/reflection in the optical transmittance and reflectance spectra, because the effective mass obtained by this analysis purely corresponds to *m*_c_^*^. In fact, *m*_c_^*^ in many semiconductors has determined by this method.

**Table 1:** Bandgap (*E*_g_) and conduction-band effective mass (*m*_c_^*^) of disordered ZnSnN_2_. ‘Exp.’ and ‘Calc.’ represent experimental and calculated values, respectively. (*m*_0_ denotes the free electron mass).

*E*_g_ [eV]	*m*_c_^*^	Ref.
0.98 (Calc.); 1.33–2.38 (Exp.)	0.12*m*_0_ (Calc.)	9
1.12 (Calc.); 2.12 (Exp.)	—	10
1.0 (Exp.)	0.5*m*_0_ (Exp.)	11
1.64–1.70 (Exp.)	—	12
0.94 ± 0.02 (Calc.)	(0.37 ± 0.05)*m*_0_ (Exp.)	This work

In addition, it has been reported that ZnSnN_2_ thin films grown by physical vapor deposition were heavily doped with oxygen (ZnSnN_2−*x*_O_*x*_), even when oxygen was not intentionally introduced into the growth chamber^4,10,11^. Since the incorporated oxygen acts as an electron donor^5,13^, unintentionally oxygen-doped ZnSnN_2_ films behave as *n*-type degenerate semiconductors with high electrical conductivity and free-electron absorption/reflection in the infrared region. Fioretti *et al*. have addressed the unintentional oxygen-doping problem to obtain nondegenerate ZnSnN_2_^14^. We can also make use of oxygen-doped ZnSnN_2_ to determine the conduction-band effective mass by analysing the free-electron absorption/reflection.

In this study, epilayers of disordered ZnSnN_2−*x*_O_*x*_ with various *x* values were grown. Detailed analysis of their optical absorption/reflection in the ultraviolet to infrared region were performed to reveal the *m*_c_^*^ and intrinsic *E*_g_ values. As a result, the *m*_c_^*^ and intrinsic *E*_g_ values were found to be (0.37 ± 0.05)*m*_0_ and 0.94 ± 0.02 eV, respectively. Herein, we describe the analysis in detail.

## Results and Discussion

### Film Growth and Structure

**Figure 2:**
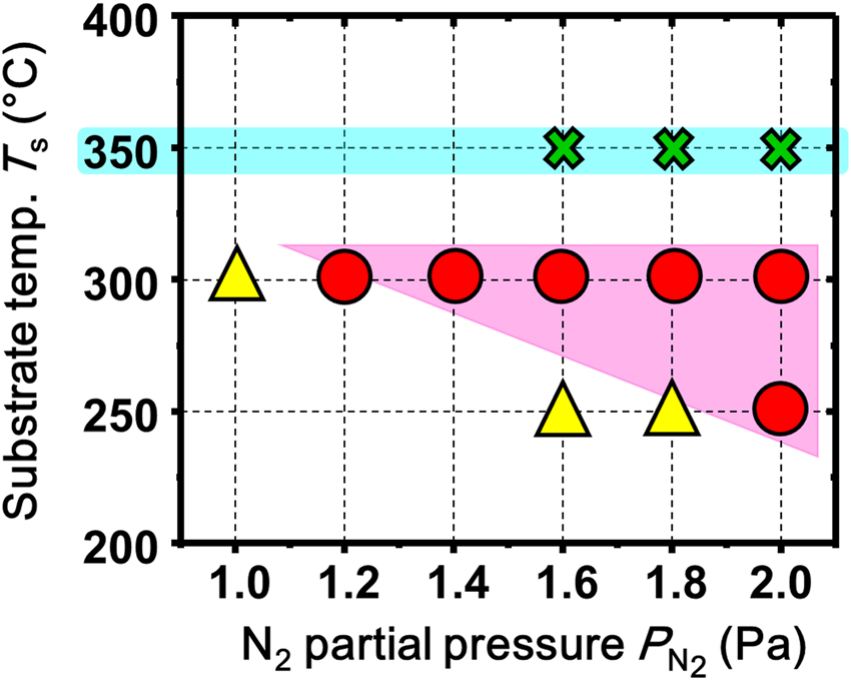
*T*_s_–${P}_{{{\rm{N}}}_{2}}$ growth parameter-based phase diagram of ZnSnN_2_ films on yttria-stabilized zirconia (111) substrates. The triangles and circles denote polycrystalline and epitaxial films, respectively. Thin films were not grown under the conditions denoted by crosses.

According to recent literature^15^, disordered ZnSnN_2_ epitaxially grows on (111) yttria-stabilized zirconia (YSZ(111)). Thus, we used YSZ(111) single-crystal substrates for film growth. The structure of the ZnSnN_2_ film strongly depended on substrate temperatures (*T*_s_) and N_2_ partial pressure (${P}_{{{\rm{N}}}_{2}}$). Figure 2 depicts the phase diagram for ZnSnN_2_ thin films, as a function of *T*_s_ and ${P}_{{{\rm{N}}}_{2}}$. As can be seen in this figure, epitaxial films were obtained in a relatively narrow region (closed circles in the shadowed region).

**Figure 3:**
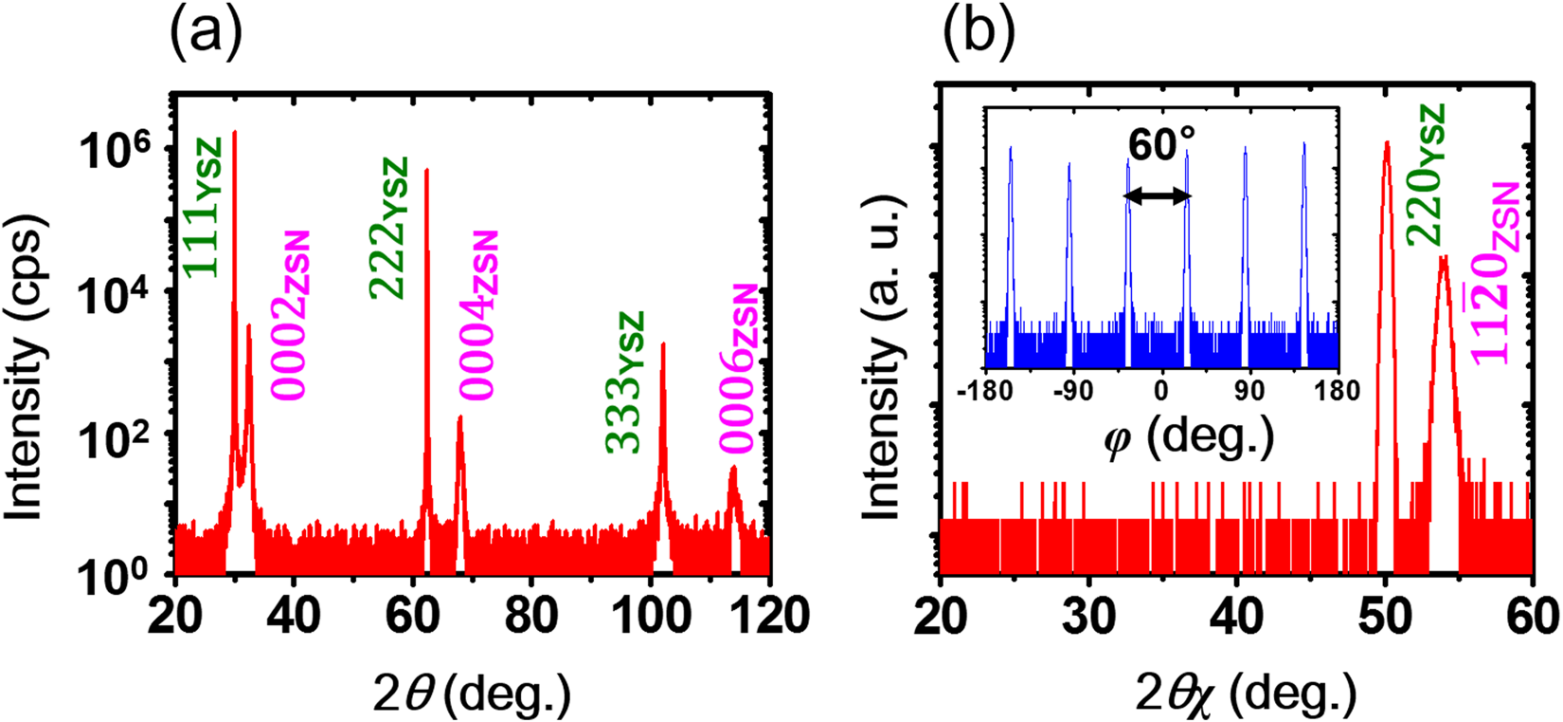
Typical X-ray diffraction patterns scanned in (**a**) *θ*-2*θ* and (**b**) *φ*-2*θ*_*χ*_ modes for ZnSnN_2_ epitaxial films. The inset of part (**b**) represents the *φ* scan of ZnSnN_2_ (11$\bar{2}$0) planes. ZnSnN_2_ is abbreviated to the ‘ZSN’ in both (**a**) and (**b**).

Figure 3a shows the out-of-plane X-ray diffraction (XRD) pattern of ZnSnN_2_ epilayers. Although the 111, 222, and 333 diffraction peaks are present in the substrate, only the 0002, 0004, and 0006 diffraction peaks from ZnSnN_2_ were observed, indicating that the (0001) planes are parallel to the (111) planes of the substrate. The ZnSnN_2_ epilayers were confirmed to be in the disordered-phase by in-plane *φ*-scans (see Methods section). Figure 3b shows a typical in-plane XRD pattern (scanned in *φ*–2*θ*_*χ*_ mode) of ZnSnN_2_ epitaxial layers. The diffraction pattern shows that the (11$\bar{2}$0) planes of the film are aligned with the (110) planes of the substrate. As shown in the inset in Fig. 3b, six evenly spaced 11$\bar{2}$0 peaks were observed in the *φ*-scan pattern, illustrating the 6-fold rotational symmetry of the basal plane in the disordered-phase (see Fig. 1b). Therefore, it can be confirmed that the disordered ZnSnN_2_ phase was grown on the YSZ(111) substrate, with the epitaxial relationship of ZnSnN_2_(0001)//YSZ(111) and ZnSnN_2_(11$\bar{2}$0)//YSZ(110).

**Figure 4:**
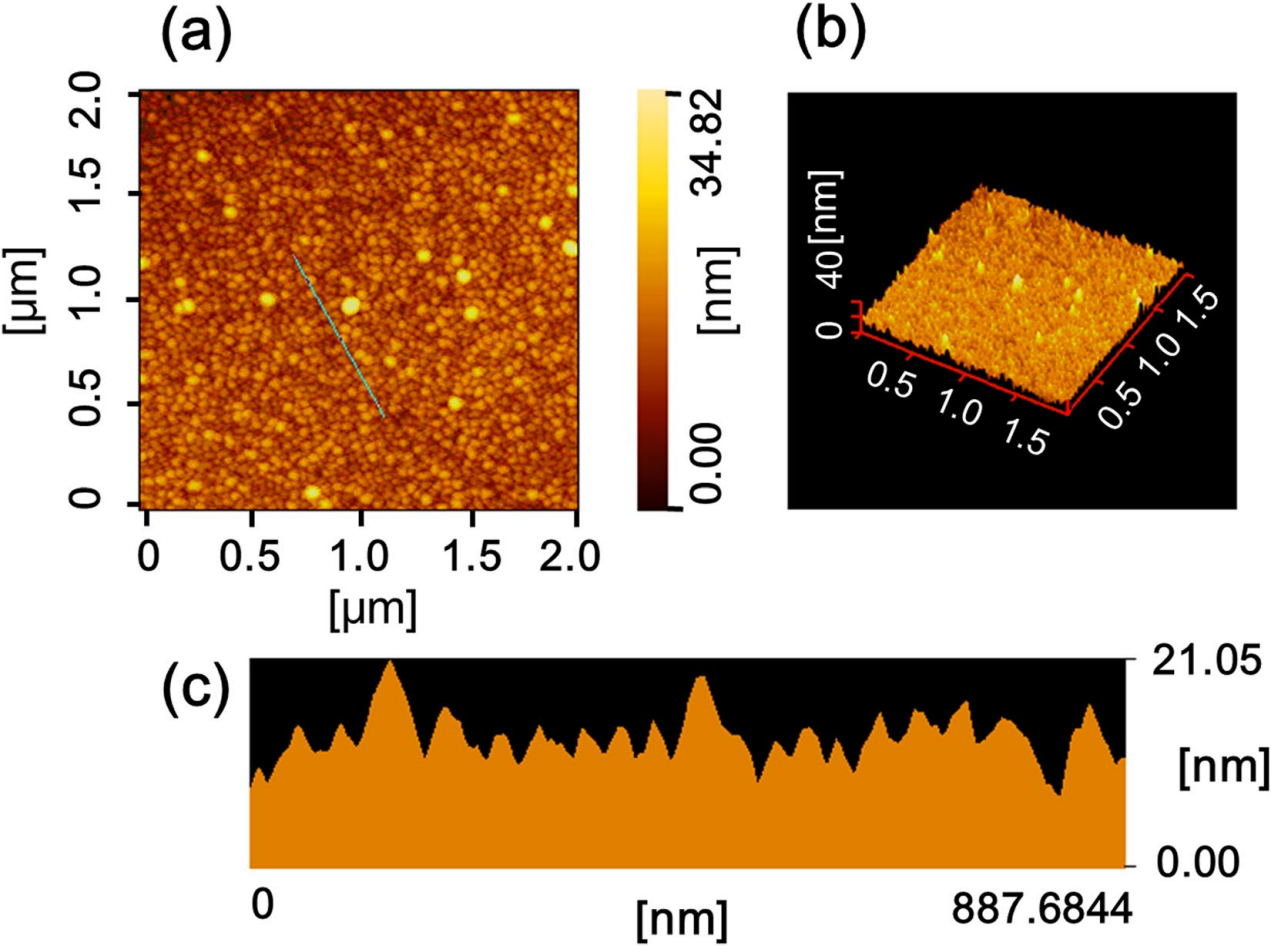
(**a**) Topographic and (**b**) three-dimensional AFM images of the ZnSnN_2_ epitaxial film surface (2 × 2 μm^2^ scan area), and (**c**) cross-sectional view along the measurement line shown in part (**a**).

Figure 4a shows the topographic atomic force microscopy (AFM) image of the ZnSnN_2_ epitaxial film. The image shows that the epitaxial film had a homogeneous compact structure comprising of small grains with lateral diameters of ~300 nm. The surface possessed a rough topography as seen from the three-dimensional image shown in Fig. 4b. The root-mean-square roughness in the 2 × 2 μm^2^ area was 1.6 nm. The rough surface is clearly illustrated in the cross-sectional image (Fig. 4c). Such rough surface may stem from the resputtering effect^16^, which is due to the bombardment of high-energy particles from the plasma. However, the surface roughness did not essentially affect the electrical and optical analyses described below.

### Film Composition and Electrical Properties

**Figure 5:**
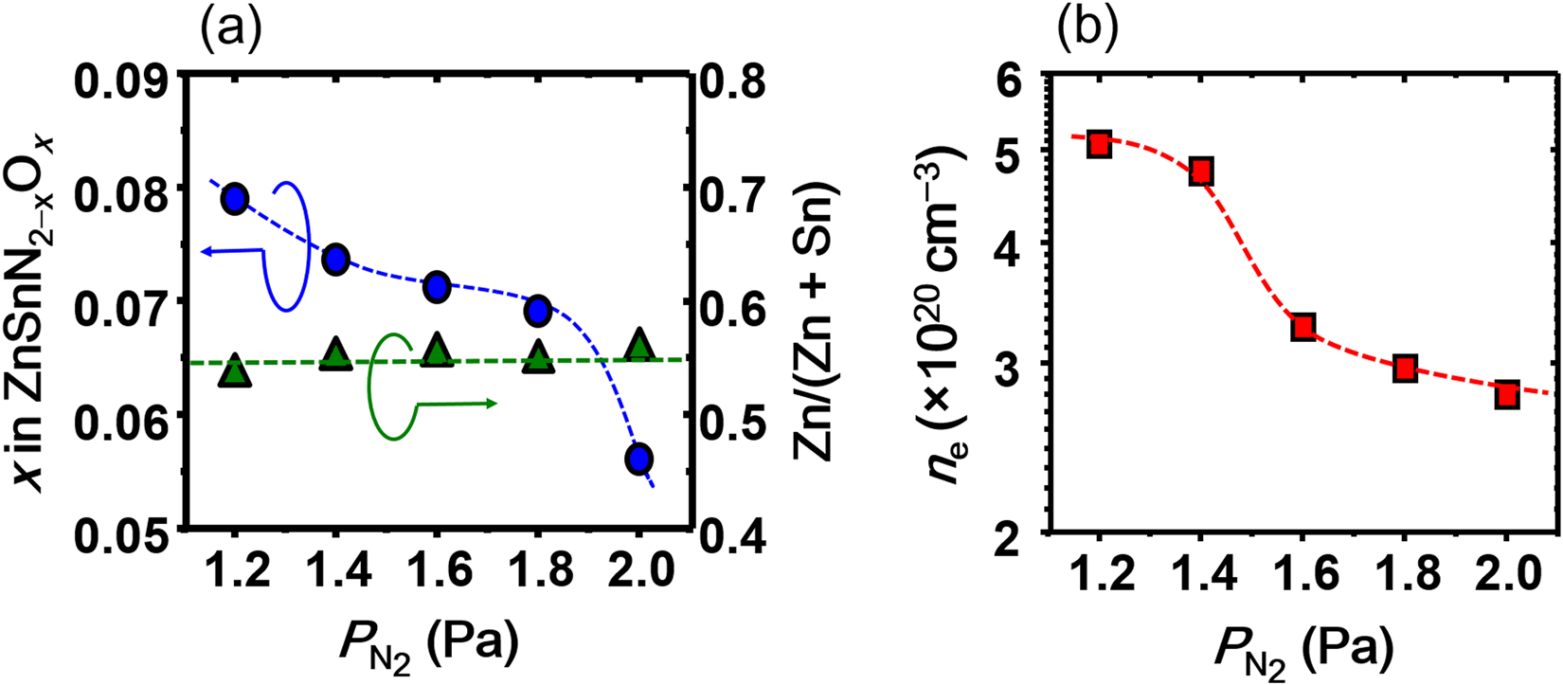
Nitrogen partial pressure (${P}_{{{\rm{N}}}_{2}}$) dependency of (**a**) oxygen concentration, *x* in ZnSnN_2−*x*_O_*x*_, (blue circles), and cation composition, Zn/(Zn + Sn), (green triangles), and (**b**) electron density, *n*_e_, plotted on a logarithmic scale. The dashed lines are visual guides.

The cationic composition, hereafter referred to as Zn/(Zn + Sn), was slightly Zn-rich in composition with Zn/(Zn + Sn) of ~0.55, which was almost independent of the growth condition (Fig. 5a). We detected oxygen in all the films, indicating that unintentional oxygen-doping occurred during the film growth (ZnSnN_2−*x*_O_*x*_). In contrast to Zn/(Zn + Sn), the oxygen concentration, *x* in ZnSnN_2−*x*_O_*x*_, depended on ${P}_{{{\rm{N}}}_{2}}$ during film growth, as shown in Fig. 5a. The *x* value decreased with increasing ${P}_{{{\rm{N}}}_{2}}$. The oxygen contamination likely originated from the residual vapor in the growth chamber. The molar ratio of water to nitrogen is expected to decrease as ${P}_{{{\rm{N}}}_{2}}$ increases or vice versa. This may be one of the reasons for the *x*–${P}_{{{\rm{N}}}_{2}}$ dependency.

All the epilayers in the present study showed *n*-type conductivity. As seen from Fig. 5b, the electron density (*n*_e_) also showed ${P}_{{{\rm{N}}}_{2}}$ dependency, as with *x*. The trend of the *n*_e_–${P}_{{{\rm{N}}}_{2}}$ dependency was very similar to that of the *x*–${P}_{{{\rm{N}}}_{2}}$, suggesting that the incorporated oxygen impurities act as electron donors similar to nitrides such as InN^17,18^, GaN^19,20^, AlN^21,22^, and Zn_3_N_2_^23,24^. Chen *et al*. theoretically predicted that oxygen impurities will occupy the nitrogen sublattice in ZnSnN_2_ and behave as single electron donors^13^. Since the *n*_e_ values were on the order of 10^20^ cm^−3^, the conduction electrons were highly degenerate (Fig. S1, Supplementary Information).

### Effective Mass

**Figure 6:**
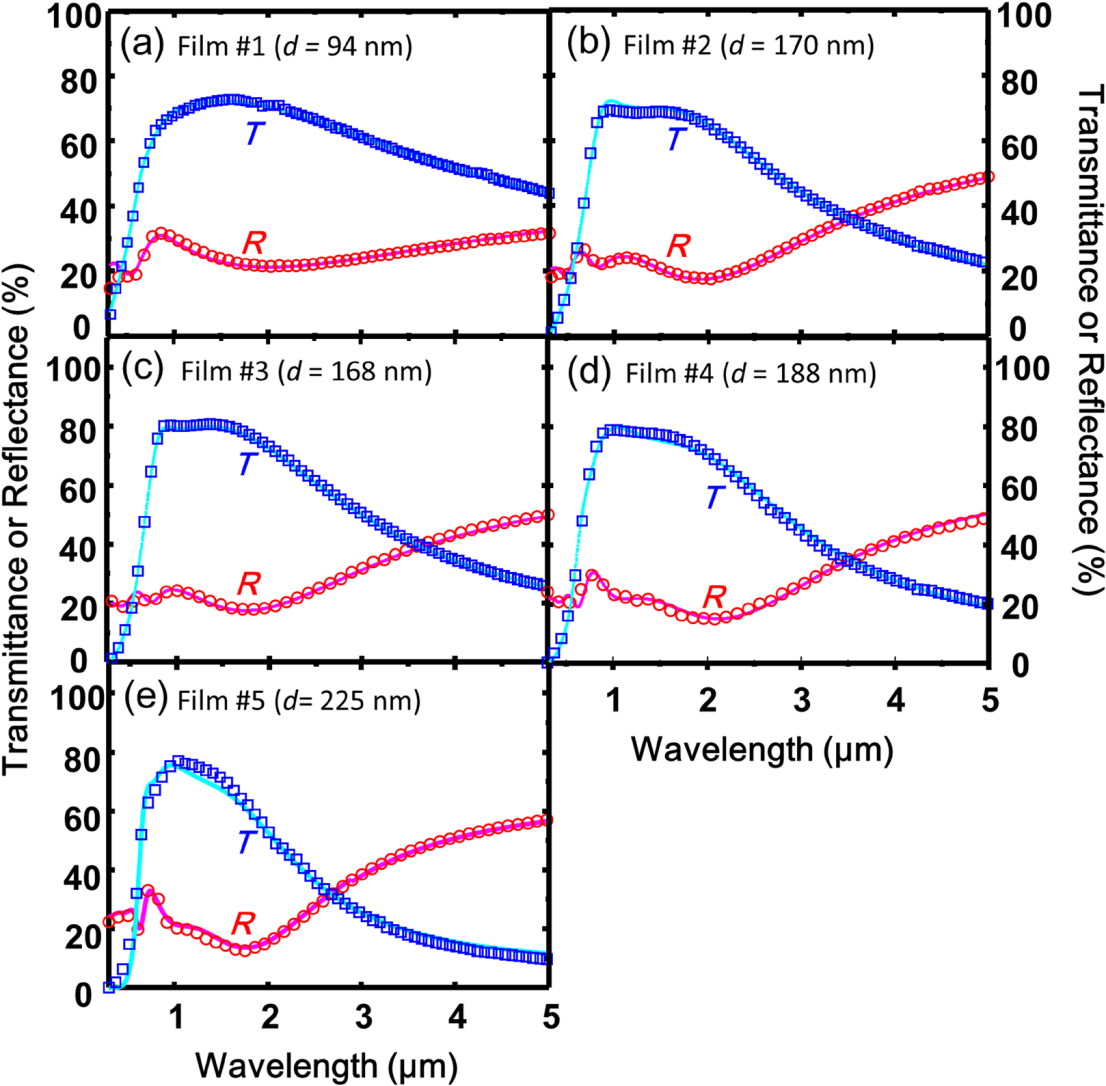
Optical transmittance (labelled by *T*) and reflectance (labelled by *R*) spectra for ZnSnN_2−*x*_O_*x*_ epilayers with different electron densities, *n*_e_: (**a**) *n*_e_ = 2.8 × 10^20^ cm^−3^, (**b**) *n*_e_ = 3.0 × 10^20^ cm^−3^, (**c**) *n*_e_ = 3.3 × 10^20^ cm^−3^ (**d**) *n*_e_ = 4.8 × 10^20^ cm^−3^, and (**e**) *n*_e_ = 5.1 × 10^20^ cm^−3^. The squares and circles represent the experimental transmittance and reflectance spectra, respectively. The solid lines are the best fit spectra theoretically calculated employing double Tauc-Lorentz dispersion model coupled with the Drude model.

Figure 6 shows the optical transmittance (*T*) and reflectance (*R*) spectra for the ZnSnN_2−*x*_O_*x*_ epilayers in the UV to IR region. In this figure, free-electron absorption/reflection can be seen. We performed a fitting analysis of the free-electron absorption/reflection using a dielectric function model to determine the conduction-band effective mass (*m*_c_^*^) of disordered ZnSnN_2_ (see Methods section).

**Figure 7:**
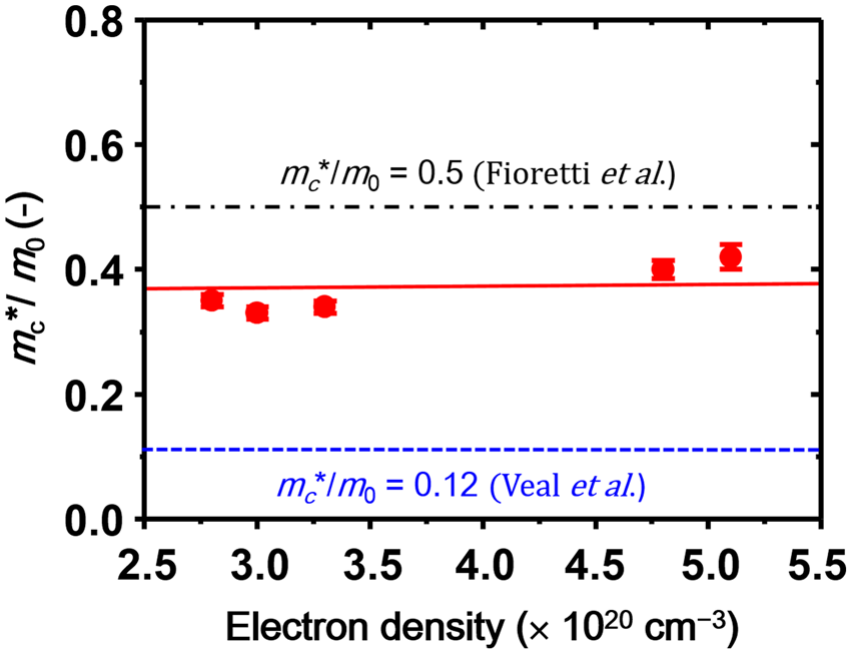
Conduction-band effective mass (*m*_c_^*^/*m*_0_) of disordered ZnSnN_2_ as a function of electron density (red circles). The solid line is a guide for the eyes. The theoretical value reported by Veal *et al*.^9^ and experimental value reported by Fioretti *et al*.^11^ are shown by the blue dashed line and black dashed-dotted line, respectively.

The square and circle marks in Fig. 6a–e represent the experimental data, and the solid lines denote the best fit spectra obtained by the double Tauc-Lorenz (TL) dispersion model coupled with the Drude model. In our combined double TL–Drude model, four fitting parameters for each TL term and two for the Drude term were considered, and described in detail in the Methods section. As can be seen in Fig. 6a–e, the model sufficiently reproduced the spectra across the entire spectral region for all the films. The values of the TL and Drude parameters obtained from the best-fit curves are summarized in Table S1 (Supplementary Information). In addition, we also show the dielectric functions obtained from the fitting analysis in Fig. S2. Using plasma energy (*E*_p_) and *n*_e_ values in Table S1, the conduction-band effective mass, *m*_c_^*^, can thus be derived (see Methods). The *m*_c_^*^ values for individual epilayers are plotted in Fig. 7, as a function of *n*_e_. The *m*_c_^*^ values ranged between (0.32–0.42)*m*_0_, where *m*_0_ denotes the free electron mass. Therefore, it was concluded that the *m*_c_^*^ value in disordered ZnSnN_2_ was (0.37 ± 0.05)*m*_0_.

First principle studies predicted *m*_c_^*^ values to be as small as ~0.1*m*_0_ in both ordered- and disordered ZnSnN_2_^4,9,25,26^. The predicted *m*_c_^*^ values are comparable to those in other III–V and Zn-based nitride semiconductors including InN (0.055*m*_0_)^27^, GaN (0.2*m*_0_)^28^, GaAs (0.083*m*_0_)^29^, and Zn_3_N_2_ (0.08*m*_0_)^30^. Although small *m*_c_^*^ values generally leads to high electron mobility (*μ)*, *μ* values in the ZnSnN_2_ epilayers have been reported to be less than or equal to 10 cm^2 ^V^−1^ s^−1^ ^9,10^. Indeed, the *μ* values of the ZnSnN_2_ epitaxial films in this study were as low as 10–20 cm^2 ^V^−1^ s^−1^. A recent experimental study by Fioretti *et al*. suggested that the *m*_c_^*^ value in disordered ZnSnN_2_ is roughly 4–5 times larger than the predicted values (~0.5*m*_0_)^11^. Our results were consistent with their value. Namely, we revealed that the *m*_c_^*^ values predicted from first-principle calculations are probably underestimated and the *m*_c_^*^ value is (0.37 ± 0.05)*m*_0_. Therefore, small electron mobility in ZnSnN_2_ can be partly explained by the somewhat large *m*_c_^*^. However, the conduction-band effective mass alone cannot fully account for the low mobility in disordered ZnSnN_2_. Further investigation of the electron transport properties is still required to understand the reasons for the low electron mobility.

Meanwhile, enhancements in *m*_c_^*^ with increasing *n*_e_ have been reported for various degenerately doped semiconductors such as GaN^31^, InN^32^, Zn_3_N_2_^33^, GaAs^34^, InSb^35^, ZnO^36,37^, TiO_2_^38^, and CdSnO_4_^39^. Such behaviours have been interpreted as a result of the nonparabolic conduction band. In contrast, the *m*_c_^*^ of ZnSnN_2_ seems to be almost independent of *n*_e_ (Fig. 7), suggesting that the conduction band of disordered ZnSnN_2_ has a parabolic shape.

### Bandgap

**Figure 8:**
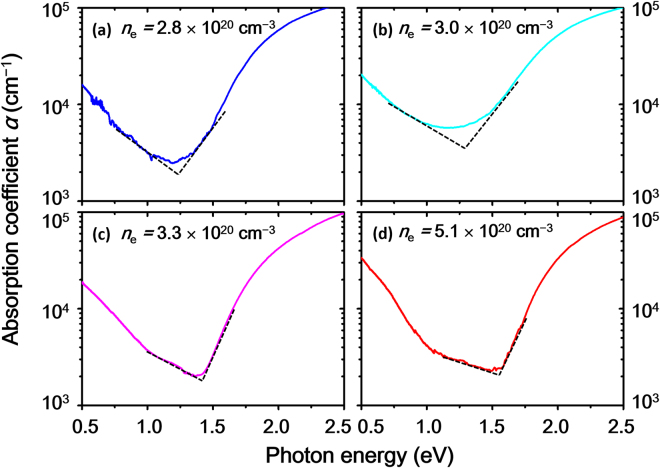
Absorption coefficient (*α*) as functions of photon energy for four selected ZnSnN_2_ films with different electron densities, *n*_e_. The photon energy at which extrapolated straight dashed lines intersect corresponds to the optical bandgap energy (*E*_g_^opt^).

The optical absorption coefficients (*α*) of the ZnSnN_2_ epilayers were calculated using the relationship *α* = *d* ^−1^ln[(1−*R*)/*T*], where *d* is the thickness of film. Figure 8 shows the absorption coefficient plotted on a log scale as a function of photon energy (*hν*) for four ZnSnN_2_ epilayers with different *n*_e_ values. The *α* spectra showed V-shaped dependency on photon energy. The absorption in the high *hν* region (*hν* > 1.5 eV) is due to the fundamental absorption, while the absorption in the low *hν* region (typically *hν* < 1.2 eV) corresponds to the free-electron absorption. We defined the optical gap energy (*E*_g_^opt^) as the photon energy where the extrapolated straight lines of the free-electron absorption and fundamental absorption intersect (see Fig. 8).

**Figure 9:**
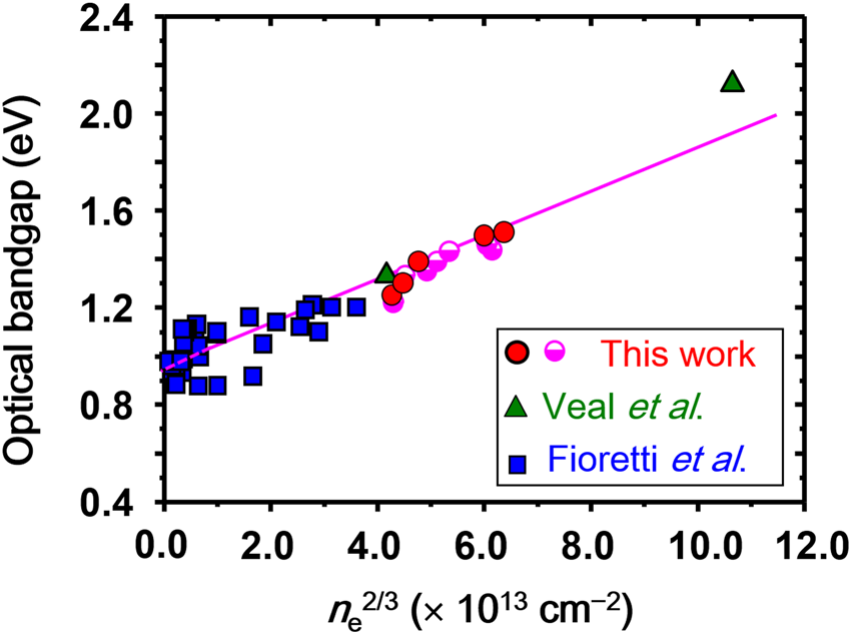
Optical bandgap of disordered ZnSnN_2_ films as a function of electron density to the power of 2/3, *n*_e_^2/3^. The circles denote the data obtained in the present study (closed circles for films in Table S1 and open circles for additional films). Data obtained from Fioretti *et al*.^11^ and Veal *et al*.^9^ are denoted as squares and triangles, respectively.

The *E*_g_^opt^ varied from 1.25 to 1.51 eV as *n*_e_ increased from 2.8 × 10^20^ to 5.1 × 10^20^ cm^−3^. Therefore, the increase in *n*_e_ caused a blue shift of the *E*_g_^opt^. The blue shift has frequently been observed in degenerate ZnSnN_2_ and is interpreted as the Burstein-Moss (BM) shift, which is a consequence of the conduction band filling effect due to heavy oxygen-doping^4,9–11^. For degenerate *n*-type semiconductors, *n*_e_-dependent *E*_g_^opt^ due to the BM effect is expressed as

1 $${E}_{{\rm{g}}}^{{\rm{opt}}}={E}_{{\rm{g}}}+{\rm{\Delta }}{E}_{{\rm{g}}}^{{\rm{BM}}}={E}_{{\rm{g}}}+\frac{{\hslash }^{2}}{2{m}_{{\rm{vc}}}^{\ast }}{(3{\pi }^{2}{n}_{{\rm{e}}})}^{2/3}$$

where *E*_g_ is the intrinsic bandgap energy and *m*_vc_^*^ is the reduced effective mass given by *m*_vc_^*^ = (1/*m*_v_^*^ + 1/*m*_c_^*^)^−1^ (*m*_v_^*^ denotes the valence-band effective mass). In heavily doped semiconductors, bandgap narrowing due to electron–electron and electron–impurity interactions also occurs: the bandgap narrowing partially compensates the bandgap widening due to the BM shift^40–42^. We calculated the bandgap narrowing due to electron–electron and electron–impurity interactions (Δ*E*_g_^e,e^ and Δ*E*_g_^e,i^, respectively) in a manner similar to the procedure described in literature^43^. Both the Δ*E*_g_^e,e^ and Δ*E*_g_^e,i^ were quite small in comparison with Δ*E*_g_^BM^, and those exhibited roughly linear dependence on *n*_e_^2/3^. Consequently, the total bandgap shift, Δ*E*_g_ = Δ*E*_g_^BM^ − Δ*E*_g_^e,e^ −Δ*E*_g_^e,i^, was almost proportional to *n*_e_^2/3^ (see Fig. S3, Supplementary Information). This result justifies plotting *E*_g_^opt^ as a function of *n*_e_^2/3^ to derive the intrinsic bandgap, *E*_g_. The *E*_g_^opt^ values for all films are plotted (closed circles) together with the values reported in literature for disordered ZnSnN_2_ films (squares^11^ and triangles^9^) as a function of *n*_e_^2/3^ in Fig. 9. Moreover, additional ZnSnN_2_ epilayers were grown under conditions similar to the films presented in Table S1. The *E*_g_^opt^ values of these additional films are also presented in Fig. 9 (open circles). As seen from Fig. 9, *E*_g_^opt^ shows linear dependency on *n*_e_^2/3^, which is consistent with the trend shown in Fig. S3. It is striking that the *E*_g_ values in this study correlate with the values reported in literature. Hence, a linear regression fit (least-square approach) was applied to all the data including the values reported in literature (Fig. 9), indicating universality within the used data, the intercept of the straight-line gives the intrinsic bandgap (*E*_g_). Accordingly, the *E*_g_ value of disordered ZnSnN_2_ was determined to be 0.94 ± 0.02 eV. The *E*_g_ value reasonably agrees with the value predicted by the first-principle study for fully disordered ZnSnN_2_ (*E*_g_ = 0.98 eV)^9^.

In the present study, we did not take the cation composition into account for the determination of *E*_g_. The cation composition may be relevant for the *E*_g_ value, though it is not clear whether the effect is significant or not. The cation composition dependency of *E*_g_ is currently a research focus for ZnSnN_2_.

## Conclusion

In summary, the conduction-band effective mass and intrinsic bandgap of disordered ZnSnN_2_ were optically determined to get an insight into the fundamental properties. Heavily oxygen-doped ZnSnN_2−*x*_O_*x*_ epitaxial layers were grown with various electron densities. These epilayers exhibited free-electron absorption/reflection in the infrared region. The detailed analysis of the free-electron absorption/reflection revealed that the conduction-band effective mass is (0.37 ± 0.05)*m*_0_. To the best of our knowledge, this study is the first to quantitatively determine *m*_c_^*^. Furthermore, the blue shift of the optical bandgap was analysed in the framework of the Burstein-Moss effect to determine the intrinsic bandgap, which has been in debate to date. Our optical bandgap values and those obtained from literature showed a universal 2/3-power dependency on the electron density. From this dependency, the intrinsic bandgap of disordered ZnSnN_2_ was determined to be 0.94 ± 0.02 eV. We believe that the findings in the present study provide important information to establish this material as a photovoltaic absorber.

## Methods

### Thin Film Growth

ZnSnN_2_ thin films were epitaxially grown on YSZ(111) single-crystalline substrates by reactive radio-frequency (RF) magnetron sputtering using a Zn_0.5_Sn_0.5_ alloy target (diameter of 10 cm and purity of 3 N), at *T*_s_ ranging from 250 to 350 °C. The base pressure of ~2 × 10^−4^ Pa was established prior to the film-growth. An RF power of 70 W was applied to the target. A mixture of Ar and N_2_ gas with various N_2_/(N_2_ + Ar) ≡ *f*(N_2_) ratios was introduced into the chamber through two independent mass flow controllers with a total flow rate of 5 sccm. The working pressure in the chamber (*P*_w_) was held at 2.0 Pa during film growth. Films were grown under ${P}_{{{\rm{N}}}_{2}}$ ranging from 1.0 to 2.0 Pa. The nitrogen partial pressure was defined as ${P}_{{{\rm{N}}}_{2}}$ = *f*(N_2_) × *P*_w_. The growth time was adjusted to obtain films with thicknesses of 100–300 nm.

### Characterization Methods

A Rigaku ATX-G X-ray diffractometer with Cu K*α* radiation was employed to perform out-of-plane (*θ*-2*θ*) and in-plane (*φ*-2*θ*_*χ*_) scans to evaluate the structure of the films. To ensure that grown ZnSnN_2_ epilayers were in the disordered-phase, we performed in-plane *φ*-scans. Provided that the ZnSnN_2_ films are in ordered-phase with the orthorhombic symmetry, a characteristic 110 peak will be observed at an in-plane diffraction angle (2*θ*_*χ*_) of 20.07° in the *φ*-scans (Fig. S4a in Supplementary Information). We carried out the in-plane *φ*-scans at the fixed 2*θ*_*χ*_ angle of 20.07° several times, but no peak was observed (Fig. S4b in Supplementary Information). Even when the 2*θ*_*χ*_ angle was slightly increased or decreased, the 110 peak did not appear. These results indicate that the ZnSnN_2_ films epitaxially grown on YSZ(111) were in the disordered-phase, which is consistent with the results reported in literature^15^. The surface morphology was characterized with an atomic force microscope (Hitachi AFM5100N). The compositions (Zn/(Zn + Sn) and *x*) of the ZnSnN_2_ films were examined by X-ray photoelectron spectroscopy (PHI Versa Probe), using monochromated Al K*α* (*hν* = 1486.6 eV) radiation. The relative sensitivity factor (RSF) approach was exploited to determine the compositions. It was confirmed that the compositions determined by the RSF method were consistent with those determined by Rutherford backscattering spectrometry^33,44^. Hence, it was concluded that the compositions in this study are sufficiently reliable. The description of the RSF approach are detailed in the Supplementary Information. Electrical properties were determined by Hall-effect measurements in the van der Pauw configuration (Toyo Corp. Resitest 8200). Optical transmittance and reflectance were collected between 0.3 and 5.0 µm using a UV-Vis-NIR spectrophotometer (Shimadzu UV-3150) and FTIR spectrometer (Shimadzu IRAffinity-1).

### Fitting Analysis of *T* and *R* Spectra

Owing to the large electron density on the order of 10^20^ cm^−3^, free-electron absorption and reflection are clearly seen in the infrared region (see Fig. 6a−e). The well-known Drude dielectric model was employed to describe the optical response by the free electrons in the IR region. The Drude function is given by,

2 $${\varepsilon }_{{\rm{D}}}(E)=-\frac{{E}_{{\rm{P}}}^{{\rm{2}}}}{{E}^{2}-i{{\rm{\Gamma }}}_{{\rm{D}}}E}$$

where *E* is the photon energy, *Г*_D_ represents the broadening parameter, and *E*_P_ denotes the plasma energy, which can be expressed as

3 $${E}_{{\rm{P}}}=\hslash {(\frac{{e}^{2}\cdot {n}_{e}}{{\varepsilon }_{0}\cdot {m}_{{\rm{c}}}^{\ast }})}^{\frac{1}{2}}$$

Here, *ħ*, *e*, and *ε*_0_, denote the reduced Planck constant, elemental charge, and the static dielectric constant of free space, respectively. As seen from Equation 3, *E*_p_^2^ is theoretically proportional to *n*_e_ when the dielectric response in the IR region can be described by the Drude model. In fact, *E*_p_^2^ showed a linear dependency on *n*_e_ (Fig. S5, Supplementary Information), suggesting appropriate use of the Drude function.

In addition to the Drude model, the Tauc-Lorentz (TL) dispersion model was considered to describe the optical response across the whole spectral region (see Fig. 6). The explicit expression of the TL model is given in literature^45^. The TL model has proved to be useful for the description of the fundamental absorption of many semiconductors^36,45–47^. Recently, Deng *et al*. demonstrated that the dielectric response of ZnSnN_2_ in UV to visible region can be reproduced by double TL functions^48^. Hence, we modelled the dielectric function of degenerate ZnSnN_2_ as a sum of double TL (*ε*_TL1_(*ω*) and *ε*_TL2_(*ω*)) and Drude functions, whereby *ε*(*ω*) = *ε*_TL1_(*ω*) + *ε*_TL2_(*ω*) + *ε*_D_(*ω*). In this study, *ε*_TL1_(*ω*) and *ε*_TL2_(*ω*) were assigned to the fundamental absorption of the bandgap and the transition that corresponds to the M_4_−M_3_ transition in group-III nitrides, respectively^49,50^. Theoretical *T* and *R* spectra calculated via the Fresnel formulas combined with the *ε*(*ω*) function were fitted to the experimental spectra. In the fitting procedure, a set of {*A*_TL_, *Г*_TL_, *E*_0_, *E*_T_} for each TL term and {*E*_p_, *Γ*_p_} for the Drude term were selected as fitting parameters. Here, *A*_TL_, *Г*_TL_, *E*_0_, and *E*_T_ in the TL term represent the oscillator strength, broadening parameter, resonance energy, and Tauc gap energy, respectively^45^.

## Electronic supplementary material

Supplementary Information
