# Supplementary material for: Conduction-band effective mass and bandgap of ZnSnN2 earth-abundant solar absorber
Source: Sci Rep. 2017 Nov 8;7:14987. doi: 10.1038/s41598-017-14850-7 (PMC5678229; doi:10.1038/s41598-017-14850-7)
Supplement: Supplementary file 1 — Supplementary Information [file 41598_2017_14850_MOESM1_ESM.doc]

Supplementary information

Conduction-band effective mass and bandgap of ZnSnN_2_ earth-abundant solar absorber

Xiang Cao^1,*^, Fumio Kawamura^2^, Yoshihiko Ninomiya^1^, Takashi Taniguchi^2^, and Naoomi Yamada^1,*^

^1^Department of Applied Chemistry, Chubu University, Kasugai, 487-8501, Japan
^2^National Institute for Materials Science (NIMS), Tsukuba, 305-0044, Japan

^*^Corresponding authors: jscaoxiang@126.com, n-yamada@isc.chubu.ac.jp

**1. Degenerate conduction electrons in ZnSnN_2−_*_x_*O*_x_***


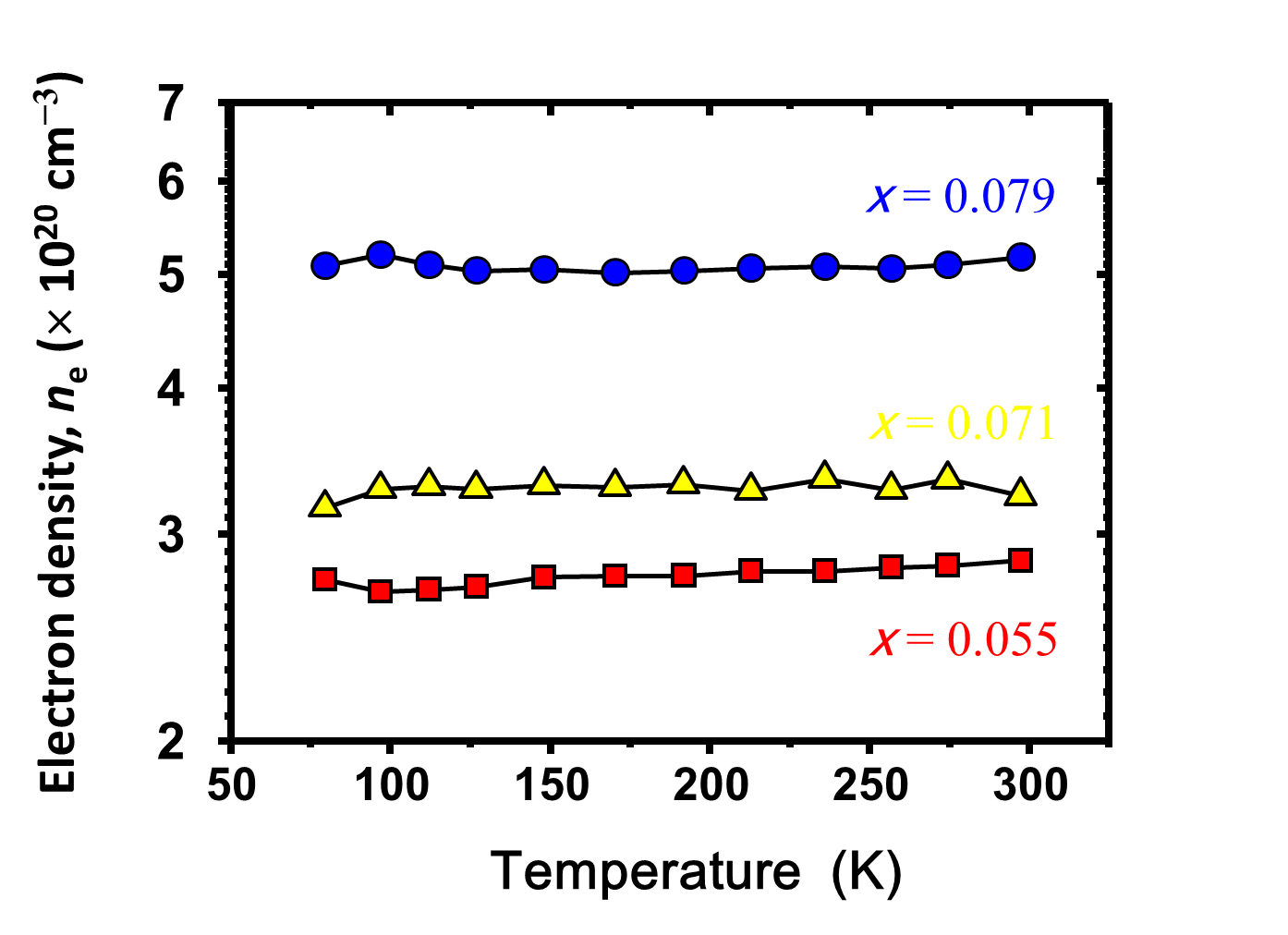


**Figure S1.** Temperature (*T*) dependence of electron density (*n*_e_) for three selected ZnSnN_2−_*_x_*O*_x_* epilayers with *x* = 0.055, 0.071 and 0.079.

**2. Double Tauc-Lorentz model parameters and real part & imaginary part of the dielectric constant**

**2.1 Double Tauc-Lorentz model parameters**

**Table S1.** Electron density (*n*_e_), Tauc-Lorentz parameters (the oscillator strength *A*_TL_, the broadening parameter *Г*_TL_, the resonance energy *E*_0_, and *E*_T_ is the Tauc gap energy) and Drude parameters (plasma energy, *E*_p_ and broadening factor, *Γ*_D_ in the Drude function) extracted from the best fit spectra shown in Figs. 6a−e in the main text. The errors were determined by several different least squares fitting runs using different initial parameters.

| **Film**  **No.** | ***n*_e_,**  **× 10^20^cm^−3^** | ***ε*_TL1_(*ω*)** | | | | ***ε*_TL2_(*ω*)** | | | | ***ε*_D_(*ω*)** | |
| --- | --- | --- | --- | --- | --- | --- | --- | --- | --- | --- | --- |
|  |  | ***A*_TL1_ [eV]** | ***Г*_TL1_**  **[eV]** | ***E*_01_**  **[eV]** | ***E*_T1_**  **[eV]** | ***A*_TL2_**  **[eV]** | ***Г*_TL2_**  **[eV]** | ***E*_02_**  **[eV]** | ***E*_T2_**  **[eV]** | ***E*_P_ [eV]** | ***Г*_D_ [eV]** |
| #1 | 2.8 | 25.40 ± 0.11 | 3.28 ± 0.01 | 2.66 | 1.24 ± 0.01 | 155.80 ± 0.17 | 14.52 | 8.35 | 2.64 | 1.04 ± 0.02 | 0.14 ± 0.01 |
| #2 | 3.0 | 23.44 ± 0.09 | 3.24 ± 0.01 | 2.66 | 1.24 ± 0.02 | 159.26 ± 0.09 | 14.52 | 8.35 | 2.64 | 1.11 ± 0.01 | 0.14 ± 0.02 |
| #3 | 3.3 | 29.88 ± 0.10 | 3.29 ± 0.01 | 2.66 | 1.34 ± 0.01 | 136.53 ± 0.13 | 14.52 | 8.35 | 2.64 | 1.15 ± 0.01 | 0.13 ± 0.01 |
| #4 | 4.8 | 25.90 ± 0.15 | 3.33 ± 0.01 | 2.66 | 1.40 ± 0.01 | 169.50 ± 0.11 | 14.52 | 8.35 | 2.64 | 1.28 ± 0.01 | 0.16 ± 0.01 |
| #5 | 5.1 | 86.94 ± 0.35 | 3.56 ± 0.03 | 2.66 | 1.69 ± 0.02 | 112.45 ± 0.22 | 14.52 | 8.35 | 2.78 ± 0.05 | 1.31± 0.02 | 0.26 ± 0.03 |

**2.2 Real and imaginary parts of dielectric function**


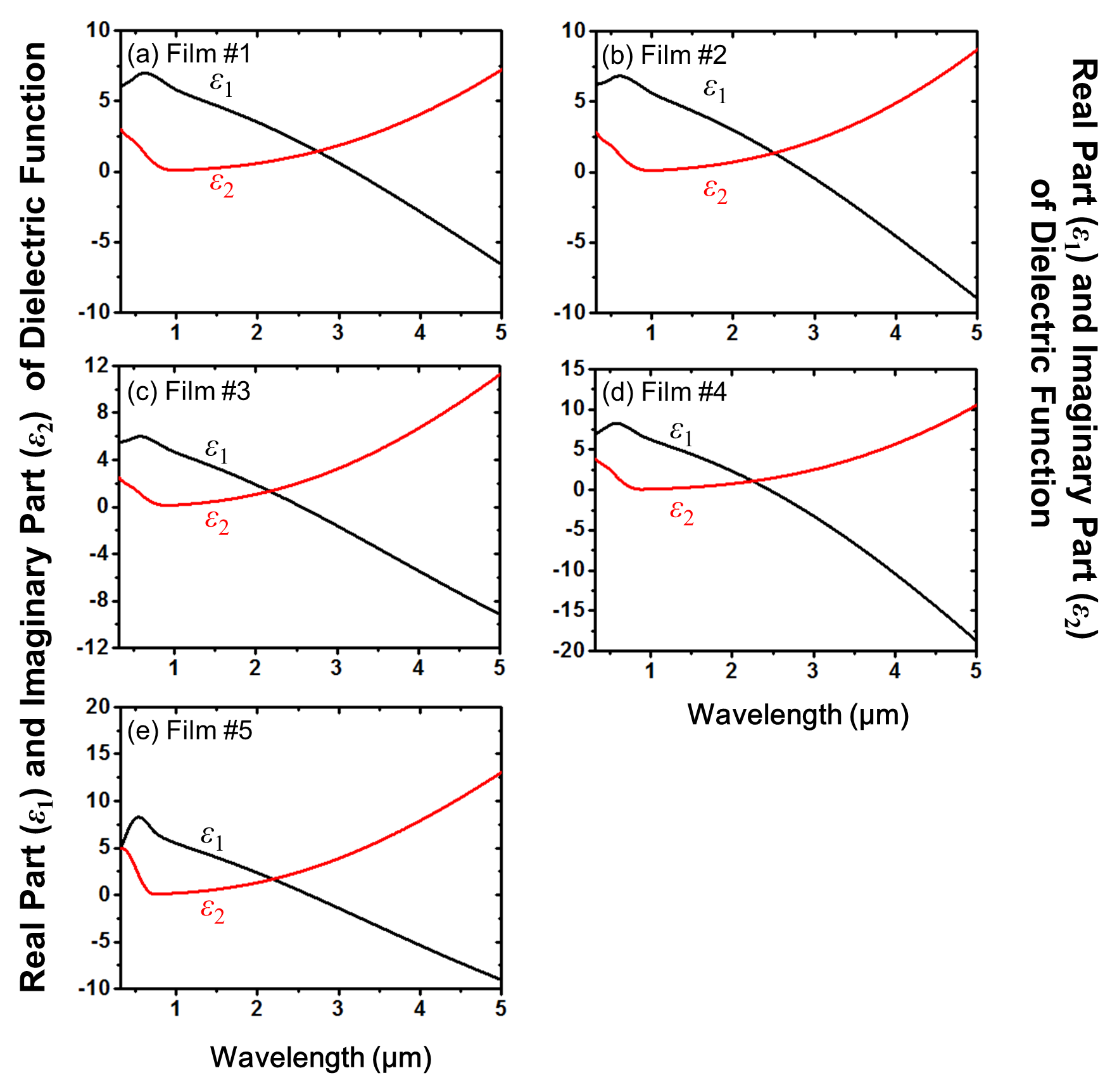


**Figure S2.** Real and imaginary parts of dielectric function (*ε*_1_ and *ε*_2_, respectively) for films #1−5.

**3. Total bandgap shift, Δ*E*_g_ = Δ*E*_g_^BM^ - Δ*E*_g_^e,e^ -Δ*E*_g_^e,i^, as a function of *n*_e_^2/3^**


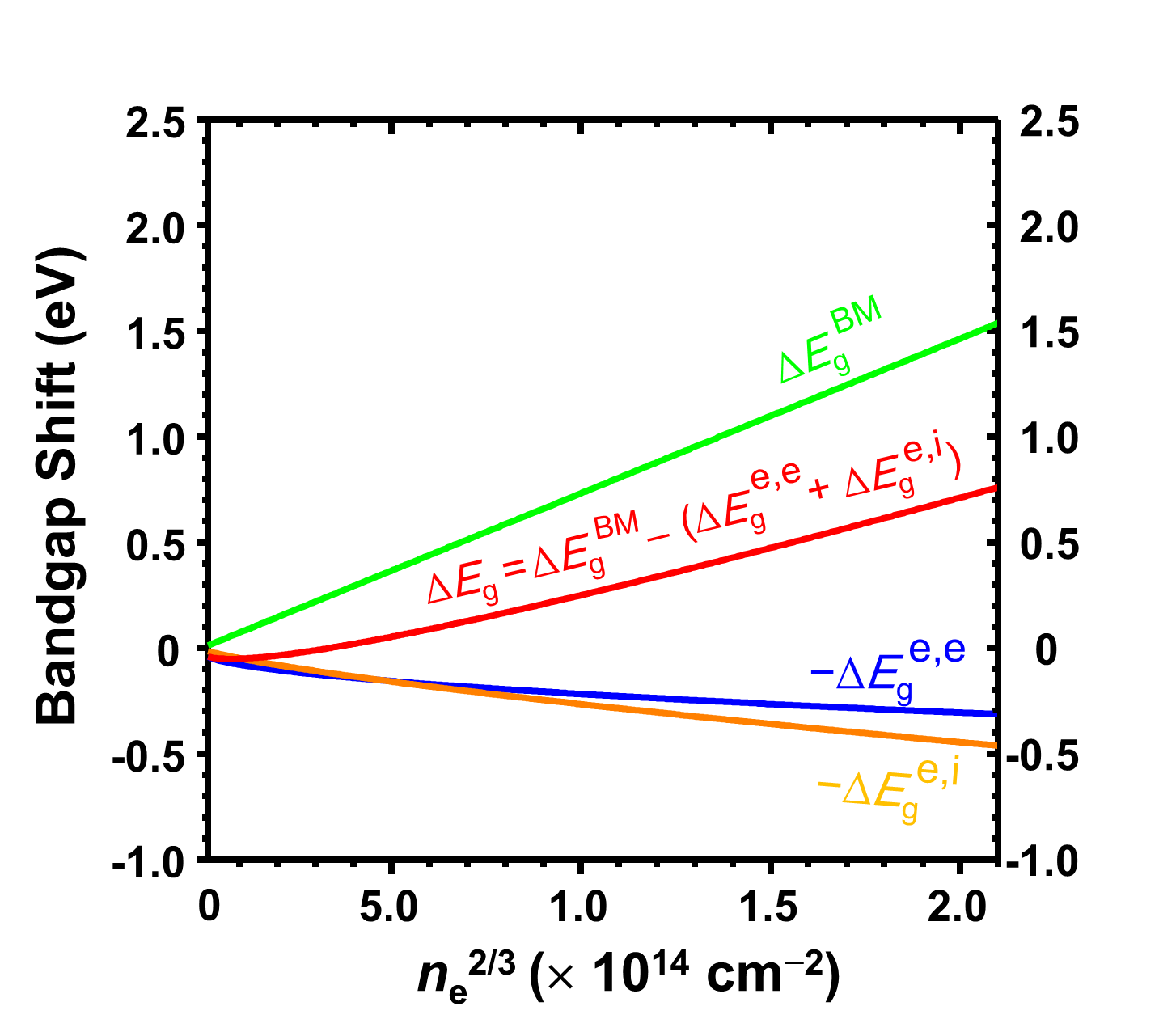


**Figure S3.** Bandgap widening due to the Burstein-Moss effect (Δ*E*_g_^BM^), bandgap narrowing due to electron-electron and electron-impurity interactions (Δ*E*_g_^e,e^ and Δ*E*_g_^e,i^, respectively), and total bandgap shift (Δ*E*_g_) as functions of electron density to the power of 2/3, *n*_e_^2/3^. Δ*E*_g_^e,e^ and Δ*E*_g_^e,i^ were calculated using the method described in Ref. 43. For the calculations, the static dielectric constant (*ε*_s_) and reduced effective mass (*m*_vc_^*^) were assumed to be *ε*_s_ = 11 (Ref. 25) and *m*_vc_^*^ = 0.5*m*_0_ (Ref. 12), respectively.

**4. 110 reflection of ordered ZnSnN_2_**


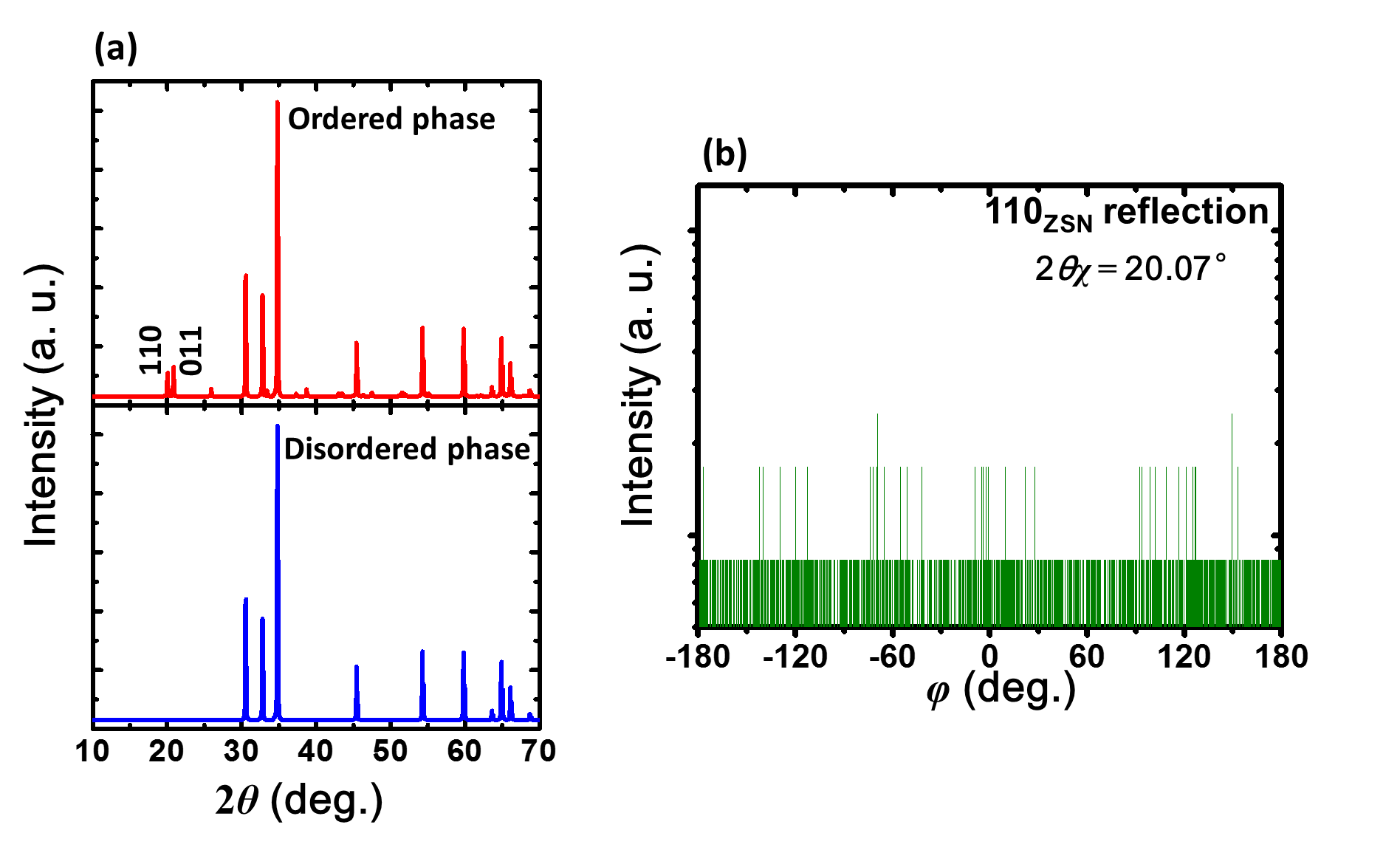


**Figure S4.** (**a**) Simulated powder X-ray diffraction (XRD) patterns for ordered-phase and disordered-phase of ZnSnN_2_, upper panel and lower panel, respectively. (**b**) XRD *φ*-scan pattern scanned at 2*θ_χ_* = 20.07° (diffraction angle of the 110 reflection in upper panel (**a**) of ZnSnN_2_ epilayers.

**5. Relative sensitivity factor (RSF) approach for the chemical composition determination**

In our present work, X-ray photoelectron spectroscopy (XPS) was performed on both as-deposited and 3-min Ar^+^-sputter-etched surface films. The latter was carried out by the sputter-etching using an Ar^+^-gun with an ion energy of 4 keV and emission current of 7 mA, with a raster size of 3 × 3 mm^2^. The semi-quantitative compositional analysis was done by fitting of the XPS core-spectra using non-linear least squares procedure: the function of the sum of Gaussian and Lorentzian was used for the peak function, and the Shirley algorithm was adopted for the background subtraction.

The relative sensitivity factor (RSF) approach was employed to determine the atomic ratio of *X_Zn_*/*X*_Sn_, which is given by

$\frac{X_{\text{Zn}}}{X_{\text{Sn}}}=\frac{I_{\text{Zn}}}{S_{\text{Zn}}}\cdot\frac{S_{\text{Sn}}}{I_{\text{Sn}}}$,

where, *I*_Zn_ and *I*_Sn_ represent the integral intensities of Zn 2p_3/2_ and Sn 3d_5/2_ peaks, respectively, and *S*_Zn_ and *S*_Sn_ respectively denote the relative sensitivity factors of Zn and Sn. The *x* values were estimated by averaging the atomic ratios of *X*_O_/*X*_Zn_ and *X*_O_/*X*_Sn_. The *X*_O_/*X*_Zn_ and *X*_O_/*X*_Sn_ are expressed as,

$\frac{X_{\text{O}}}{X_{\text{Zn}}}=\frac{I_{\text{O}}}{S_{\text{O}}}\cdot\frac{S_{\text{Zn}}}{I_{\text{Zn}}}$,

and

$\frac{X_{\text{O}}}{X_{\text{Sn}}}=\frac{I_{\text{O}}}{S_{\text{O}}}\cdot\frac{S_{\text{Sn}}}{I_{\text{Sn}}}$,

where *I*_O_ and *S*_O_ represent the integral intensity of O 1s peak and the sensitive factor of oxygen, respectively. We used *S*_Zn_ = 3.354, *S*_Sn_ = 4.095, and *S*_O_ = 0.711 as provided in PHI^1^.

1. Moulder, J. F., Stickle, W. F., Sobol, P. E. & Bomben, K. D. *Handbook of X-ray photoelectron spectroscopy* (Minnesota 1995).

**6. Validity check of the plasma energy, *E*_p_**


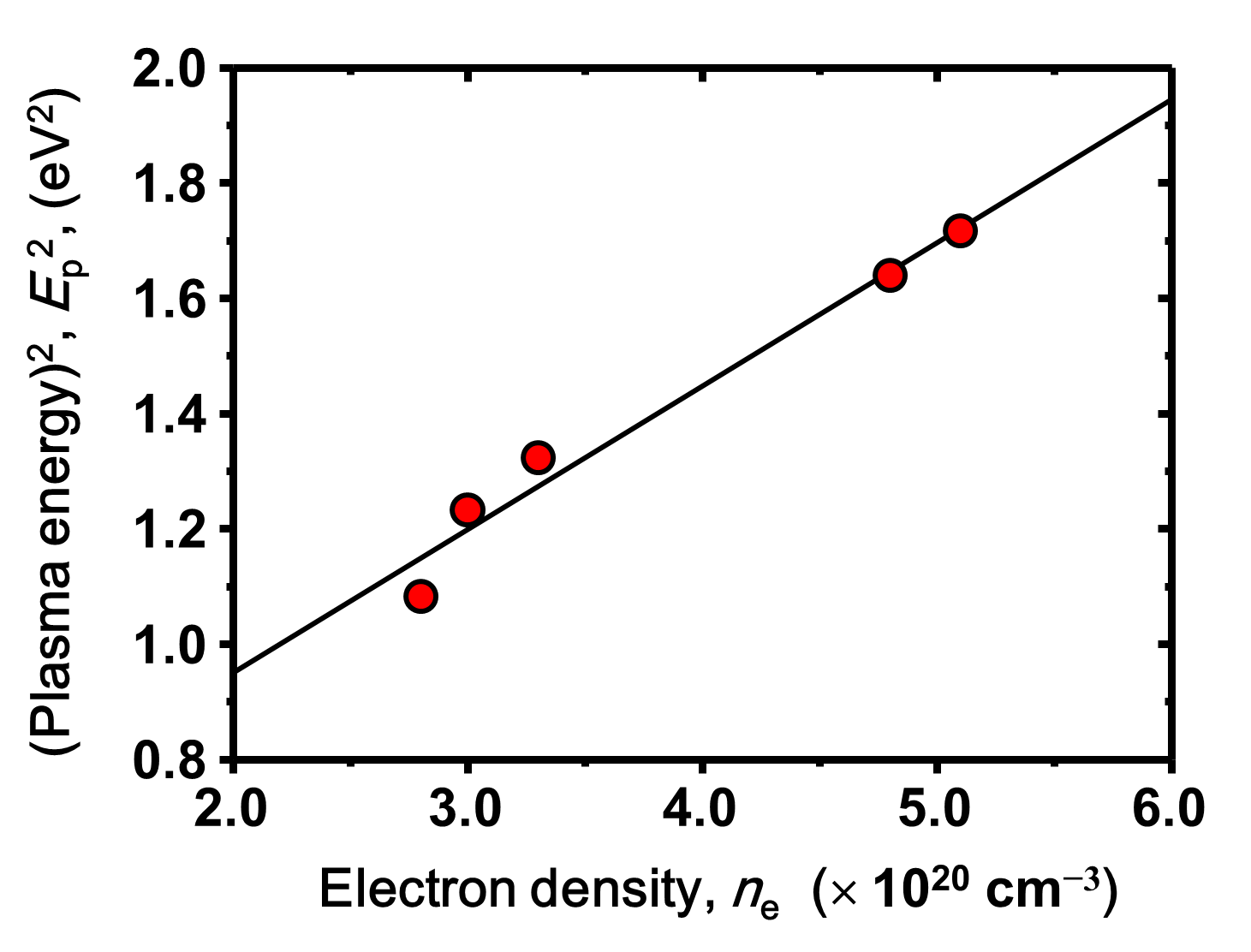


**Figure S5.** Squares of plasma energy (*E*_p_^2^) as a function of electron density (*n*_e_). A linear dependence of *E*_p_^2^ on *n*_e_ is clearly seen, indicating that *E*_p_^2^ is proportional to *n*_e_ as described in Equation 3 in the main text.
